# A Fairness of Data Combination in Wireless Packet Scheduling

**DOI:** 10.3390/s22041658

**Published:** 2022-02-20

**Authors:** Sovit Bhandari, Navin Ranjan, Yeong-Chan Kim, Pervez Khan, Hoon Kim

**Affiliations:** 1 IoT and Big-Data Research Center, Incheon National University, Yeonsu-gu, Incheon 22012, Korea; sovit198@gmail.com (S.B.); ranjannavin07@gmail.com (N.R.); kyc0288@inu.ac.kr (Y.-C.K.); 2 Department of Electronics Engineering, Incheon National University, Yeonsu-gu, Incheon 22012, Korea; pervaizkanju@hotmail.com

**Keywords:** artificial intelligence, ethical, fairness, packet scheduling, wireless networks, convolutional neural network

## Abstract

With the proliferation of artificial intelligence (AI) technology, the function of AI in a sixth generation (6G) environment is likely to come into play on a large scale. Moreover, in recent years, with the rapid advancement in AI technology, the ethical issues of AI have become a hot topic. In this paper, the ethical concern of AI in wireless networks is studied from the perspective of fairness in data. To make the dataset fairer, novel dataset categorization and dataset combination schemes are proposed. For the dataset categorization scheme, a deep-learning-based dataset categorization (DLDC) model is proposed. Based on the results of the DLDC model, the input dataset is categorized based on the group index. The datasets based on the group index are combined using various combination schemes. Through simulations, the results of each dataset combination method and their performance are compared, and the advantages and disadvantages of fairness and performance according to the dataset configuration are analyzed.

## 1. Introduction

### 1.1. Motivation

With the rapid growth and proliferation of artificial intelligence (AI) technologies and applications, the question of ethical AI has arisen. In recent years, a large number of works have been intensively studied on the principles and guidelines of AI ethics. AI fairness is one of the key elements of the principles or guidelines, and data fairness in AI learning is considered as one of the main concerns [1]. The work in [2] offers a new approach: a set of principled solutions based on the emerging and exciting science of socially aware algorithm design. The authors explain how we can better embed human principles into machine code—without halting the progress of data-driven scientific exploration. The work in [3] examined several real-world applications that exhibited biases in different ways and listed several sources of biases that can affect AI applications. The authors also created a taxonomy for fairness definitions that machine learning researchers have defined to avoid the existing biases in AI systems.

Since the sixth generation (6G) wireless network system is likely to integrate AI technologies into its system [4], packet scheduling is one of the most important areas of the wireless system where the ethical aspects of AI should be considered, as it is responsible for selecting user equipment (UEs) to transmit data packets. The goal of packet scheduling in a wireless network system is to ensure efficient and fair resource utilization for all UEs [5,6,7]. Some of the popular traditional approaches to packet scheduling in wireless networks include: round-robin (RR), maximum system throughput (MST), and proportional fair scheduling (PFS) [8,9,10,11].

In the RR-based scheme, UEs are selected in a round-robin fashion so that all UEs have the equal probability of being selected in the time domain despite radio channel status. This type of scheme cannot perform adaptive adjustment according to change in channel condition. To address the drawback of RR, MST was proposed. In an MST-based scheme, the UEs with the best channel are selected to maximize the total system throughput in each scheduling epoch. This strategy sacrifices the fairness among UEs because those UEs with poor channel conditions have high likelihood of not being selected. To address the drawbacks of both RR- and MST-based schemes, PFS was proposed by F. Kelly [10]. PFS, a widely used scheduling scheme, provides a tradeoff between overall system throughput and fairness among UEs selection. The works described in [8,9,10,11] are iterative-based schemes, which are more difficult to implement in a real-time network with a large number of system dynamics, since they require a comparatively high computational effort [12].

In recent years, there has been major breakthroughs in the field of wireless communications using AI technology [13,14,15]. Some of the works which have considered AI to solve packet scheduling problems in wireless network systems are listed in [16,17,18,19]. In [16], a support vector machine (SVM) model-based packet scheduling in wireless communication networks has been proposed. The SVM model is fed on the data produced by the PFS scheme considering only two UE scenario, which is not practical for real life implications. In [17], a supervised deep neural network (DNN)-based method is proposed to schedule channel in interference-limited wireless networks. The DNN is fed on the data produced by the iterative-based sequential convex approximation (SCA) algorithm. In [18], the joint subcarrier and power allocation problem is studied, where a deep Q-network is employed for scheduling decision making and a DNN is trained for power allocation using supervised learning. In [19], deep reinforcement learning (DRL)-based double-deep Q network (DDQN) framework is proposed to make scheduling decisions in the edge computing environment. However, the works mentioned in [16,17,18,19] more focused on increasing overall system throughput and did not guarantee the selection of UEs having low channel strength.

Motivated by the growing interest in ethical AI [1,2,3], in order to guarantee fairness in the UEs selection procedure for packet scheduling while maximally maintaining existing wireless system performance, a novel dataset categorization and dataset combination schemes are proposed in this paper.

### 1.2. Related Work

In this sub-section, we present some of the related work that deals with maintaining as well as investigating the fairness of data in the AI domain and are listed in [20,21,22,23,24,25,26,27].

In [20], the fairness of the recidivism prediction instrument is discussed and shows the differential impact of using a recidivism prediction instrument that is not known to have prediction errors. In [21], a regression-based approach is presented to remove the implicit bias present in the correctional offender management profiling for alternative sanctions (COMPAS) dataset. The COMPAS dataset includes suspect records from Broward County with information on their jail and prison time, demographics, criminal history, and COMPAS risk scores [28]. Similarly, in [22], an unbiased regression-based approach is presented to predict the actual assessed targets by ensuring impartiality in terms of gender and race.

In [23], a data profiling framework based on deep learning and statistical model algorithms is proposed to improve the quality of data by finding outliers. This framework was validated using a salary dataset published by the state of Arkansas. In [24], a counterfactual fairness framework that uses tools of causal inference to ensure group fairness (treating different groups equally) is presented. In [25], a criterion for discrimination against a specified sensitive attribute in supervised learning is proposed to predict some target based on available features. The framework is tested on the problem of fairly predicting the acceptance of law students. In addition, in [26,27], an AI-based real-time toolkit for fairness assurance is presented.

In [26], the Aequitas toolkit is presented, which allows users to test models with respect to various bias and fairness metrics for different population subgroups. In [27], the AI Fairness 360 (AIF360) toolkit developed by IBM is presented. This toolkit helps transfer fairness research algorithms into an industrial setting and provides a benchmark for fairness algorithm evaluation. Moreover, it also provides a platform for fairness researchers to share their ideas.

The work presented in [20,21,22,23,24,25,26,27] addressed fairness with respect to various demographic groups, which cannot be used to ensure fairness in wireless data because they do not contain demographic information. Inspired by the group fairness shown in [20,21,22,23,24,25,26,27], this work proposes a wireless dataset D categorization and combination scheme to ensure fairness in selecting UEs for packet transmission at any scheduling epochs.

### 1.3. Contribution and Organization

In this paper, a wireless dataset D categorization scheme and various combination schemes are proposed to give user candidates with poor channel status the same opportunity as user candidates with good/medium channel status. The contribution of this paper can be summarized as follows:

1. In the first part, a deep learning (DL)-based data categorization (DLDC) scheme is proposed to categorize the wireless dataset D, which contains the timestamp, information about the connecting UEs, information about the base transceiver station (BTS), and information about the channel capacity between the connecting UEs and the BTS, into three different groups. The categorization is performed on the basis of the channel capacity between the connecting BTS and UEs.
2. In the second part, different data combination schemes are proposed to combine the group-index-based categorized wireless dataset D using a DLDC scheme such that the UEs having poor channel capacity are also included for the data transmission process. In the data combination schemes, different methods are proposed to show how the UEs selection process is affected when there is an imbalance in the dataset so that we can recognize the importance of the ethical dataset.

The remainder of this paper is organized as follows: Section 2 describes the schemes for categorizing and combining wireless dataset D. Section 3 introduces the DLDC scheme. Section 4 evaluates the performance of the proposed DLDC model, and based on which the results of the data combination schemes are shown. Section 5 discusses the overall results and limitations of this work. Finally, in Section 6, conclusions are drawn.

## 2. Dataset Categorization and Dataset Combination Schemes

**Figure 1:**
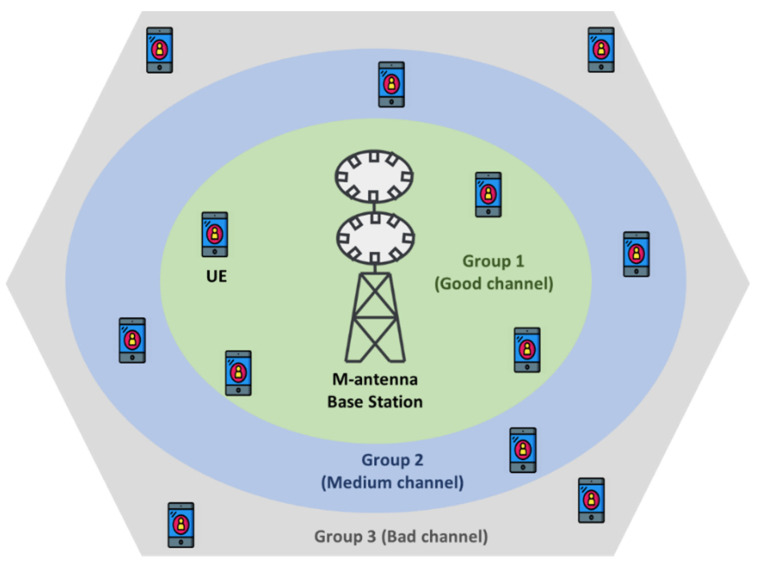
A wireless system environment containing *M*-antenna BTS serving *N_s_* UEs out of *N_a_* UEs.

In this section, wireless system dataset categorization and combination schemes are proposed to maintain fairness in UE selection while maintaining system throughput for data transmission in the next scheduling epoch. To propose a fairness in UE selection procedure for packet transmission, a wireless system environment consisting of M-antenna BTS is considered as shown in Figure 1 to create the wireless system dataset. Since fairness can be modeled in the resource-deficit scenario, in the figure, it is shown that the single BTS can serve $N_{s}$ UEs out of $N_{a}$ UEs at any time instance t.

### 2.1. Dataset Categorization Scheme

**Figure 2:**
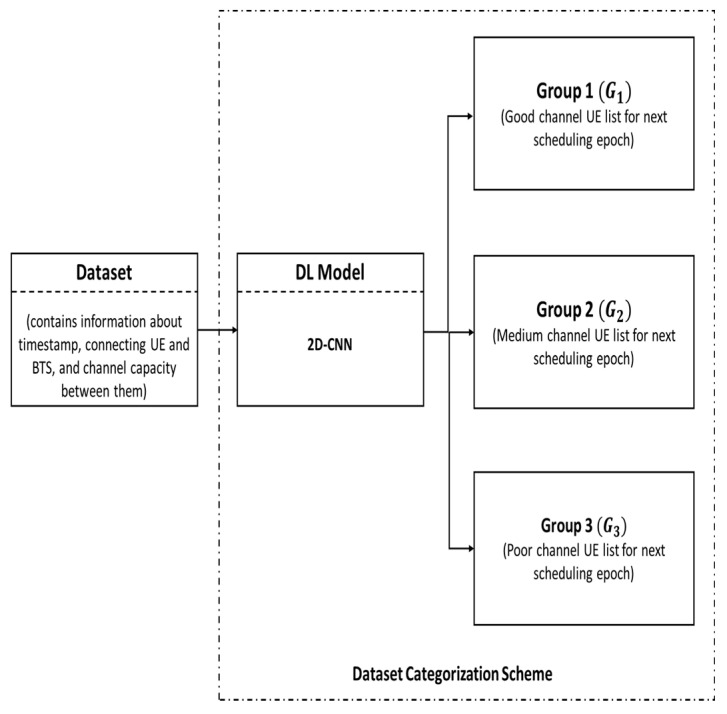
A wireless system dataset categorization scheme based on DLDC.

In this part, the wireless dataset D obtained from the system environment is divided into three different groups, namely, Group 1 ($G_{1}$), Group 2 ($G_{2}$), and Group 3 ($G_{3}$), as shown in Figure 1. Since the UEs are constantly moving in the wireless system environment, the UEs that belong to one group in one time period may belong to another group in another time period. Therefore, AI is required to determine the group index $G_{i}$ (i∈{1, 2, 3}) of the connecting UEs instantaneously. Since the 6G wireless system is likely to embed AI functions into its system, in this paper, the need for AI is satisfied by the DLDC model, which is a DL-based 2D convolutional neural network (CNN) model. The categorization of the wireless dataset D on the basis of group index $G_{i}$ can be performed by employing it to the trained DLDC model, as shown in Figure 2.

In Figure 2, the category $G_{1}$ dataset contains channel information of the connecting UEs that exhibit good channel strength while connecting to the M-antenna BTS at any time instance t. Similarly, the category $G_{2}$ dataset contains channel information of the connecting UEs that have medium channel strength while connecting to the M-antenna BTS at any time instance t. Likewise, the category $G_{3}$ dataset contains channel link information of the connecting UEs that have poor channel strength while connecting to the M-antenna BTS at any time instance t.

The detailed explanation of the DLDC scheme to categorize the dataset into multiple groups is discussed in Section 3.

After the categorization of the dataset containing the different channel strength information between the connecting UEs and M-antenna BTS into three groups, the multiple dataset combination schemes are provided in the next sub-section to maintain the UEs selection fairness while maintaining overall system throughput.

### 2.2. Dataset Combination Schemes

**Figure 3:**
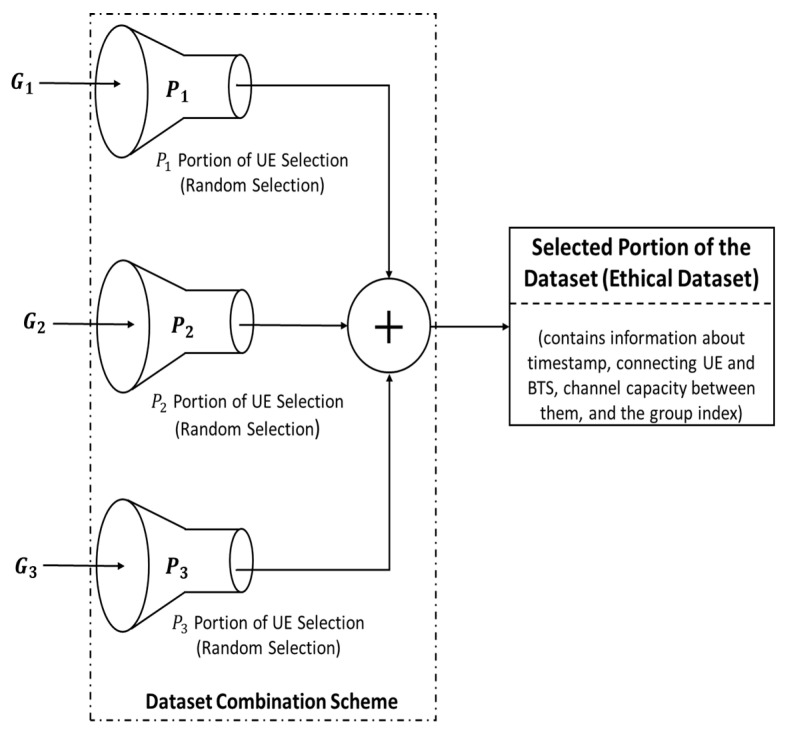
A general wireless system dataset combination scheme.

In this sub-section, the dataset obtained from the dataset categorization scheme, i.e., $G_{1}$, $G_{2}$, and $G_{3}$, is merged using different dataset combination schemes (see Figure 3) so that the fairness of UE selection is maintained.

In Figure 3, only a selected portion of the dataset from each group is combined to meet the requirements of the wireless system environment and to make the data more ethical. As shown in the figure, part $P_{1}$ of the $G_{1}$ dataset, part $P_{2}$ of the $G_{2}$ dataset, and part $P_{3}$ of the $G_{3}$ dataset are randomly selected before they are combined. This ensures that the UEs from each group are given priority for data transmission despite the channel strength, provided it meets the minimum requirements for data transmission.

Based on the values of the selection portion $P_{1}$, $P_{2}$, and $P_{3}$, the dataset combination schemes $S_{k}\;\left(k\in \left\{1,\;2,\;3\right\}\right)$ are classified into three different schemes, namely, the random dataset combination scheme (RDCS) or Scheme 1 ($S_{1}$), the equal dataset combination scheme (EDCS) or Scheme 2 ($S_{2}$), and the weighted dataset combination scheme (WDCS) or Scheme 3 ($S_{3}$), as shown in Figure 3.

**Figure 4:**
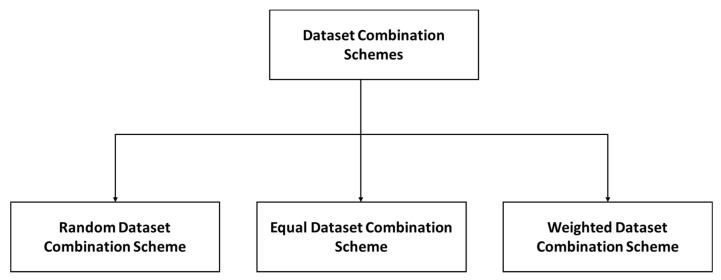
Types of wireless dataset combination schemes.

Based on the values of the selection portion $P_{1}$, $P_{2}$, and $P_{3}$, the data combination scheme can be further divided into the three different schemes, namely, random dataset combination scheme (RDCS), equal dataset combination scheme (EDCS), and weighted dataset combination scheme (WDCS), as shown in Figure 4.

The different kinds of dataset combination schemes are briefly described in the sub-sections of this part.

#### 2.2.1. Random Dataset Combination Scheme

In this scheme, the dataset containing the radio link information of the connecting UEs and the BTS is randomly combined without any group information index. The fairness in the selection of UEs is not guaranteed in this scheme.

#### 2.2.2. Equal Dataset Combination Scheme

In this scheme, an equal proportion of the dataset from each group is used before combination, i.e., the ratio of $P_{1}$:$\;P_{2}$:$\;P_{3}$ is set to 1:1:1. In this scheme, fairness is ensured in the selection of UEs, since the UEs with the medium and poor channel strength are also preferred in the data transmission process.

#### 2.2.3. Weighted Dataset Combination Scheme

In this scheme, an unequal proportion of the dataset from each group is used before the combination, e.g., the ratio of $P_{1}$:$\;P_{2}$:$\;P_{3}$ is set to 1:2:3. If the ratio of $P_{1}$:$\;P_{2}$:$\;P_{3}$ is set to 1:2:3, it means that the candidate of Group 3 related to weak channel strength will receive higher priority in data transmission compared to the other groups.

## 3. Deep-Learning-Based Dataset Categorization Scheme

In this section, the DLDC scheme, a 2D CNN-based DL model, is proposed to categorize datasets into multiple groups. The dataset with input features such as the timeslot, information about connecting UEs, the connecting BTS, and the channel capacity between connecting UEs, and BTS is fed into the DLDC scheme to categorize the dataset into multiple groups. Before applying DLDC to categorize the dataset, the dataset is generated for the DLDC scheme. The dataset generation procedure is described in Section 3.1.

### 3.1. Dataset Generation

In this sub-section, we create a dataset D that will be used for the DLDC model. Based on the system model shown in Figure 1, a dataset is created. It is assumed that there are 12 UEs that attempt to connect to the M-antennas BTS at each time t. The link capacity between the UEs and the BTS is determined by the Shannon capacity limit, i.e., $B\times log_{2}\left(1+SIR\right)$, where B is the channel bandwidth and SIR is the signal-to-interference ratio. It is assumed that the channel distribution of the air interface link is a Rayleigh distribution. Since large dataset is required for training the supervised DL-based model [29], the 0.1 million (M) timeslot is considered to generate moderately large dataset [30] for the 12 UEs scenario to avoid the computational effort. In [31,32], it is reported that the maximum downlink spectral efficiency for 5G is 30 bps/Hz, and for 6G, it will be 100 bps/Hz. Based on [31,32], we assumed the average SIR of our wireless system environment to be 33 dB when creating the 0.1 M 2D dataset. Since we considered the supervised 2D CNN-based learning method, the label in the dataset is created based on the value of SIR. If the SIR of the channel is greater than 33 dB, it is considered to be a good channel, so the UEs under this group are labelled as $G_{1}$. If the SIR of the channel is less than 27 dB, it is considered to be a bad channel, and the UEs under this group are called $G_{3}$. The channel that lies between the good and the bad channel is called the middle channel and is represented by $G_{2}$. The $G_{1}$, $G_{2}$, and $G_{3}$ labels are identified by the three-bit combination, i.e., [1,0,0], [0,1,0], and [0,0,1], respectively. The three-bit combination is obtained by using one-hot encoding method [33]. The simulation parameters used to create the dataset are given in Table 1.

### 3.2. Model Implementation

In DLDC scheme, we have used 2D-based CNN model for feature extraction from input dataset D. The main goal of this model is to categorize input dataset into $G_{1}$, $G_{2}$, and $G_{3}$ groups, respectively, based on the input features such as timeslot, user information, and channel capacity. Since we have only assumed single BTS in our system, the information regarding BTS is avoided from the dataset while training the model.

In the DLDC scheme, we have used a 2D-based CNN model for feature extraction from the input dataset D. The main objective of this model is to categorize the input dataset D into $G_{1}$, $G_{2}$, and $G_{3}$ groups, based on the input features such as the timeslot, user information, and channel capacity. Since we have only assumed a single BTS in our system, the information regarding BTS is avoided from the dataset while training the model.

**Figure 5:**
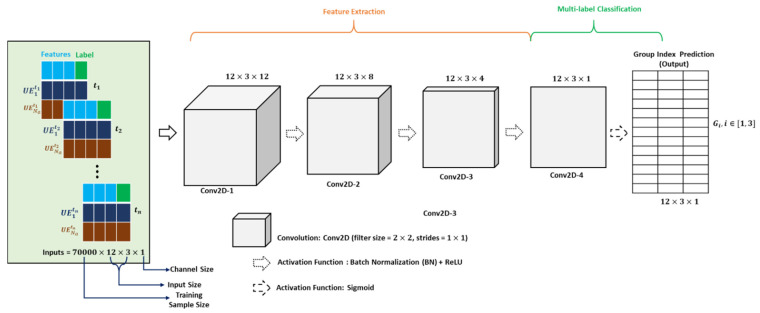
DLDC model for categorization of dataset into different groups.

The input data per timeslot are converted to the 2D format having the dimensions of 12×3×1 (height × width × channel) before employing it to the DLDC model. The height in the data depends upon the number of UEs, and the width represents the number of the input features (i.e., 3). The 70% of D, i.e., 0.07 M data of size 12×3, is used for training the model, while 20% of D (0.02 M) is used for validation. The remaining 10% of D (0.01 M) is used for testing the trained model. The 2D CNN model used in this paper is shown in Figure 5.

In Figure 5, the architecture of the DLDC model is formed by stacking four convolutional layers. First, the 2D input of size 12 × 3 (row number × column number) is used in the first 2D convolutional layer of the DLDC model shown to produce an output of size 12 × 3 × 12. In the first convolutional layer, the input is scaled up using 12 filters of size 2 × 2 with a stride of 1 × 1. In the convolved output of the first layer, batch normalization (BN) is used, and then it is passed to the rectified linear unit (ReLU). In our DLDC model, BN is used to stabilize the learning process and reduce the number of epochs required to train the neural networks [34], and the ReLU activation function is used to increase the nonlinearity of the input data and solve the vanishing gradient problem [35]. The output of the ReLU activation function of the first convolution layer is then used in the second convolution layer with filter size eight for reduction. In this layer, the size is reduced to 12 × 3 × 8. In the convoluted output of the second convolution layer, BN is applied and then fed to the ReLU activation. The output of the ReLU activation function of the second convolution layer is fed to the third convolution layer. Since only four filters are used in the third convolution layer, the output of the third convolution layer is reduced to 12 × 3 × 4. BN is applied to the output of the third convolution layer, and then passed to the ReLU activation function. The output of the ReLU activation function in the third layer is used in the last convolution layer. In the last layer, the input is convolved to produce an output of size 12 × 3 × 1 by using only one filter in this layer. Since our problem is a multi-label classification problem, the binary cross-entropy (BCE) loss function is used for training the model along with sigmoid activation function in the last convolution layer [36]. The output of our last layer is sent to the sigmoid activation function. The sigmoid activation function classifies our input data into multiple labels, i.e., $G_{1}$, $G_{2}$, and $G_{3}$, respectively.

In our DLDC scheme, we trained 0.07 M data of size 12 × 3 to achieve the multi-label classification with better accuracy. Moreover, the Adam optimizer is used to update the weight and learning rate values as it is straightforward to implement, computationally efficient, and has little memory requirements [37].

The detailed structure of our DLDC model is shown in Table 2.

## 4. Performance Evaluation

In this section, the simulation results are shown to evaluate the performance of our proposed DLDC scheme, as well as dataset combination methods such as RDCS, EDCS, and WDCS. The key performance index (KPI) of the proposed DLDC scheme is presented in terms of complexity analysis, training accuracy, test accuracy, BCE loss in the training phase, and BCE loss in the test phase. Based on the results of the DLDC scheme, RDCS, EDCS, and WDCS are applied. The KPI of RDCS, EDCS, and WDCS is shown in terms of the user selection count, fairness in user selection, and average user throughput.

To train the DLDC model, we used the Keras library on top of the TensorFlow framework in Python 3.7 as a programming platform. The training process for our datasets is performed by using a computation server (MiruWare, Seoul, Korea). The specification of the computational server includes; one Intel Core i7 CPU, four Intel Xeon E7-1680 processors, and 128 GB random access memory. The results are obtained by using a computer with 16 GB random access memory and an Intel Core i7-8700 processor. The results are used to apply different data combination schemes.

### 4.1. DLDC Model KPI

In this part, the DLDC model in terms of training accuracy, test accuracy, BCE loss in the training phase, and BCE loss in the test phase is presented. The proposed 2D CNN-based model is trained using 0.07 M 2D data. Likewise, the validation of the trained model is performed on the 0.02 M 2D data. Finally, the trained model is tested on 0.01 M 2D data. The simulation parameter used while training the model is summarized in Table 3.

**Figure 6:**
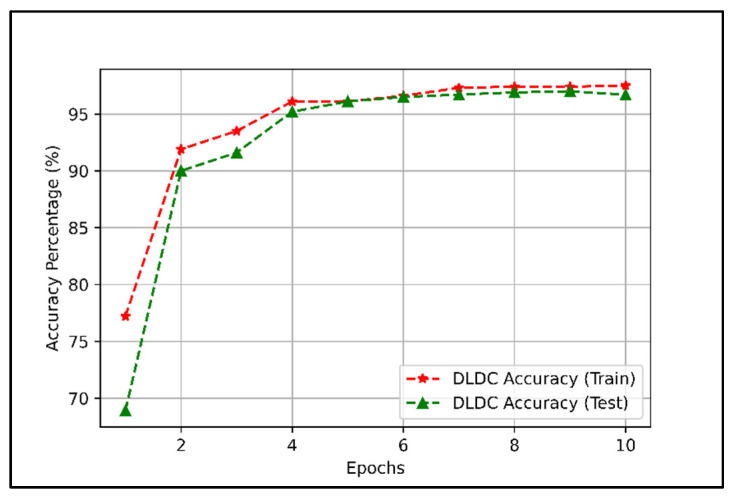
DLDC model training as well as testing accuracy for 10 training epochs.

The training accuracy and test accuracy of the trained DLDC scheme are shown in Figure 6.

Figure 6 shows that the training accuracy of the DLDC model is 97.5%, and the test accuracy is 96.7% when the model is trained for 10 training epochs. From Figure 6, we can infer that the test accuracy of the DLDC model approaches the training accuracy when the model is trained for up to 10 training epochs and settles at 96 to 97 (%).

**Figure 7:**
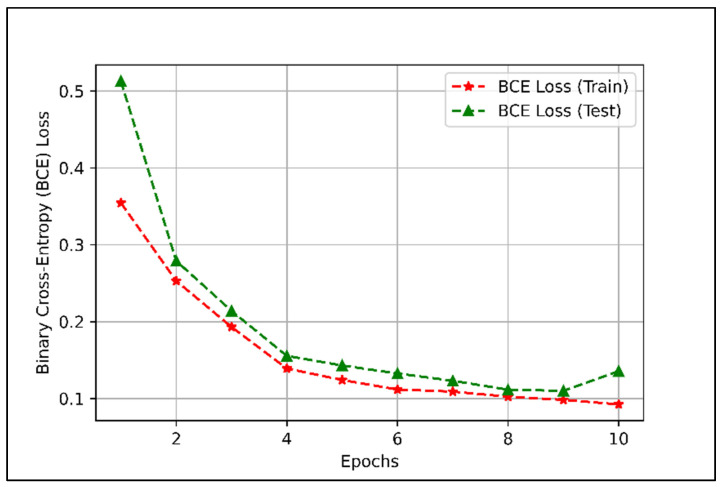
DLDC model training as well as testing binary cross entropy loss for 10 training epochs.

The training BCE loss and test BCE loss of the trained DLDC scheme are shown in Figure 7.

Figure 7 shows that the training BCE loss of the DLDC model is 0.0923, and the test BCE loss is 0.1355 when the model is trained for 10 training epochs. From Figure 7, we can infer that the test BCE loss of the DLDC model approaches the training BCE loss when the model is trained for up to 10 training epochs and settles at 0.1 to 0.15.

From Figure 6 and Figure 7, we can conclude that the DLDC model is well trained and can be further used for the categorization of dataset D in terms of group index $G_{i}$.

### 4.2. Data Combination Schemes KPI

In this part, the performance of the RDCS, EDCS, and WDCS is shown in terms of complexity analysis, user selection count, user selection fairness, and average throughput based on the group-index based dataset, i.e., $G_{1}$, $G_{2},$ and $G_{3}$, provided by the DLDC scheme.

Fairness in the selection of UEs in a wireless system environment for packet scheduling comes into play when the number of UEs attempting to connect to the BTS is greater than the BTS can actually serve in any scheduling epoch. Thus, to create a scenario that addresses the problem of fairness in the selection of UEs in the wireless system environment, the simulations assume that the BTS with M antennas can serve only 6 UEs in any scheduling epoch and that the number of UEs attempting to connect to the BTS at each time t is twice the number of UEs that the BTS can actually serve, i.e., 12. Since the size of the 2D training dataset for the DLDC model depends on the number of UEs attempting to connect to the BTS, a smaller number of UEs is assumed to avoid computational overhead in creating the wireless dataset D as well as training the DLDC model.

The channel bandwidth of the subcarriers is assumed to be 20 MHz. The minimum required data volume is assumed to be the same for all users in a given timeslot. The performances of the proposed data selection schemes are provided in terms of the 100 scheduling epochs.

#### 4.2.1. Complexity Analysis

In this part, we compare the complexity of RDCS, EDCS, and WDCS. Computational complexity is defined as the number of complex operations. The complex operations considered in the complexity analysis are complex addition, complex multiplication, and complex division [38]. The complexity of our proposed methods for combining datasets is shown in Table 4.

From Table 4, we can see that the time complexity for RDCS, EDCS, and WDCS is same, i.e., $O\left(TN_{a}\right)$.

#### 4.2.2. User Selection Count

**Figure 8:**
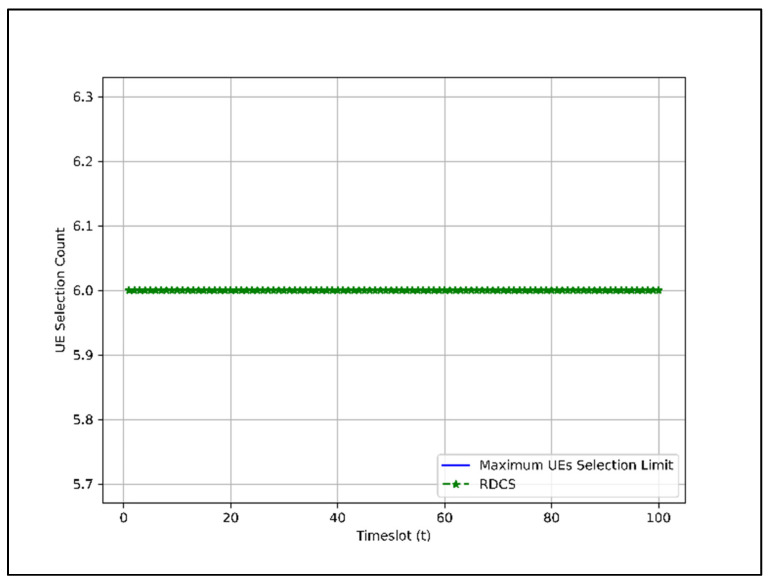
Total UEs selection count under RDCS for 100 timeslots.

For RDCS, the role of group index $G_{i}$ does not come into play, so the data extraction proportion, i.e., $P_{1}$, $P_{2}$, and $P_{3}$. Under RDCS, the UEs are assigned randomly. Based on the given simulation parameters, the total UEs that can be selected under RDCS for 100 is shown in Figure 8.

In Figure 8, the total number of UEs selected at each timeslot is six. Since the BTS can serve six UEs at each timeslot, we can say that under RDCS, the UEs slot is fully utilized.

**Figure 9:**
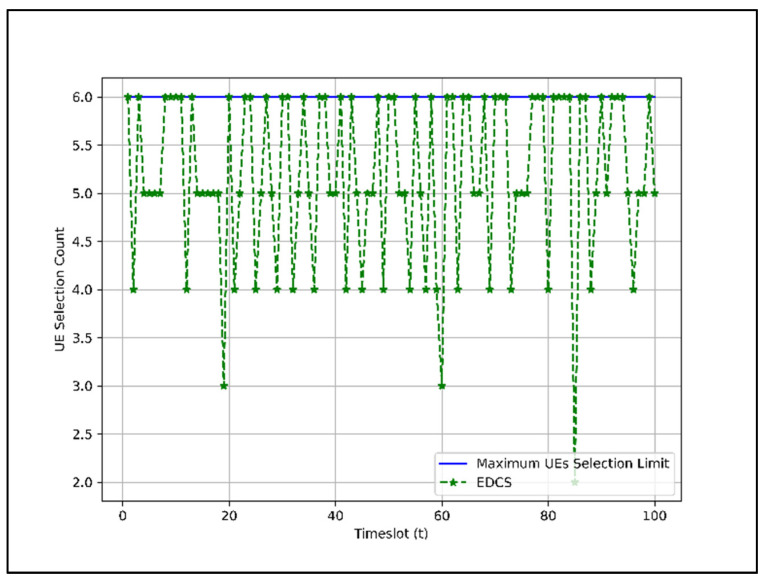
Total UEs selection count under EDCS for 100 timeslots.

For EDCS, the group index is taken into account. For each group, a maximum of two users are allowed to connect. This is because our assumed system can serve six UEs simultaneously. The total number of users that can be served by this scheme for each scheduling epoch is shown in Figure 9. In Figure 9, the number of UEs selected for packet transmission is either five or six in most timeslots, four in a few timeslots, and three or two in rare cases.

**Figure 10:**
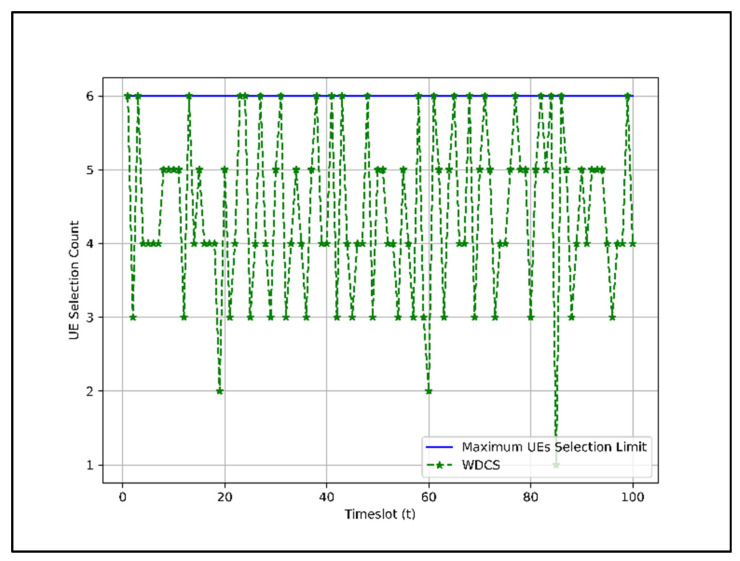
Total UEs selection count under WDCS for 100 timeslots.

In WDCS, the group index is also considered. However, in this scheme, an unequal proportion of UEs from each group is selected before combination. In our case, we set the UEs selection limit for $G_{1}$ to 1, for $G_{2}$ to 2, and for $G_{3}$ to 3, since the maximum connection limit in each time instance is set to 6. The candidate of $G_{3}$ receives a higher priority than the other groups. The total number of users that can be served by this scheme for each scheduling epoch is shown in Figure 10.

In Figure 10, the number of UEs selected for packet transmission is three to six in most timeslots, and rarely, the number of UEs selected is one or two.

In EDCS and WDCS, the UEs slots in most time instances are not as heavily occupied as in RDCS because there are fewer or no UEs in groups $G_{2}$ and $G_{3}$ in those time instances despite higher preference. By comparing Figure 8, Figure 9 and Figure 10, we can say that the RDCS is better in terms of UEs selection count.

#### 4.2.3. User Selection Fairness

**Figure 11:**
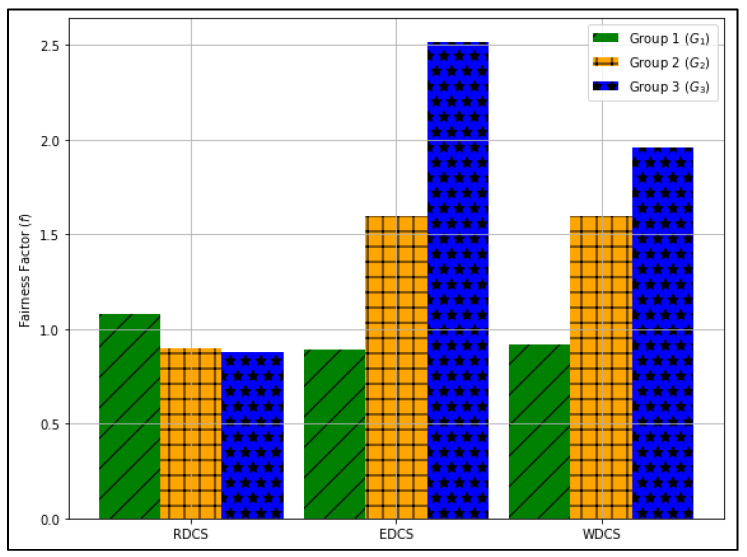
The fairness factor (f) for user selection for each group index $G_{i}$ under dataset combination schemes such as RDCS, EDCS, and WDC in 100 timeslots.

In this part, the $G_{i}$ based user selection fairness factor (f) for each dataset combination schemes $S_{k}$ is calculated as following:

(1) $$f_{G_{i}}^{S_{k}}=\frac{Portion\;of\;UEs\;selected\;for\;each\;group\;G_{i}\;under\;scheme\;\;S_{k}\;\;}{Probability\;of\;UEs\;selection\;for\;each\;group\;G_{i}\;under\;scheme\;\;S_{k}}$$

where the portion of UEs selected for each group $G_{i}\;\mathrm{under}\;\mathrm{scheme}\;S_{k}$ refers to the ratio of the total selected UEs for the data transfer process belonging to the group $G_{i}$ under scheme $S_{k}$ to the total available UEs belonging to the same group under the same scheme in a time period T. The fairness factor (f) for user selection calculated for different dataset combinations such as RDCS, EDCS, and WDCS based on the group index $G_{i}$ is shown in Figure 11.

In Figure 11, we can see that for RDCS, the values of f for $G_{1}$, $G_{2}$, and $G_{3}$ are 1.08, 0.89, and 0.87, respectively. In RDCS, the probability of selecting UEs is set to 0.5 because only 6 UEs out of 12 UEs are allowed for the data transmission process in each scheduling epoch. Since the value of f for $G_{1}$ is greater than one, while the value of f for $G_{2}$ and $G_{3}$ is less than one, RDCS is more suitable for selecting $G_{1}$ UEs.

Likewise, we can see that for EDCS, the values of f for $G_{1}$, $G_{2}$, and $G_{3}$ are 0.88, 1.59, and 2.51, respectively. In EDCS, the probability of selecting UEs belonging to $G_{1}$, $G_{2}$, and $G_{3}$ is set to 0.33 for all groups because the same proportion of UEs is assigned to each group. Since the value of f for $G_{2}$ and $G_{3}$ is greater than one while the value of f for $G_{1}$ is less than one, EDCS is more suitable for the selection of $G_{2}$ and $G_{3}$ UEs. In this scheme the value of f for $G_{1}$ is less than one because of the higher counts of UEs belonging to $G_{1}$ and comparatively lesser slot allocation for $G_{1}$.

Similarly, we can see that for EDCS, the values of f for $G_{1}$, $G_{2}$, and $G_{3}$ are 0.91, 1.59, and 1.96, respectively. In WDCS, the probability of selecting UEs belonging to $G_{1}$, $G_{2}$, and $G_{3}$ is set to 0.16, 0.33, and 0.5, respectively, as the proportion of selecting UEs is set to 1:2:3. In this scheme, the value of f for $G_{1}$ and $G_{2}$ is almost identical to the corresponding groups in EDCS, while the value of $G_{3}$ is lower than that of the corresponding group in EDCS. Despite the higher slot allocation for $G_{3}$ UEs in WDCS for packet transmission, the value of f for $G_{3}$ in WDCS is lower than the corresponding group in EDCS due to the smaller number of UEs belonging to $G_{3}$.

When we compare the results of RDCS, EDCS, and WDCS in Figure 11, we can say that EDCS is fairer than the other two schemes. If the UEs with medium/bad channel is the priority, then EDCS is the best approach.

#### 4.2.4. Average User Throughput

**Figure 12:**
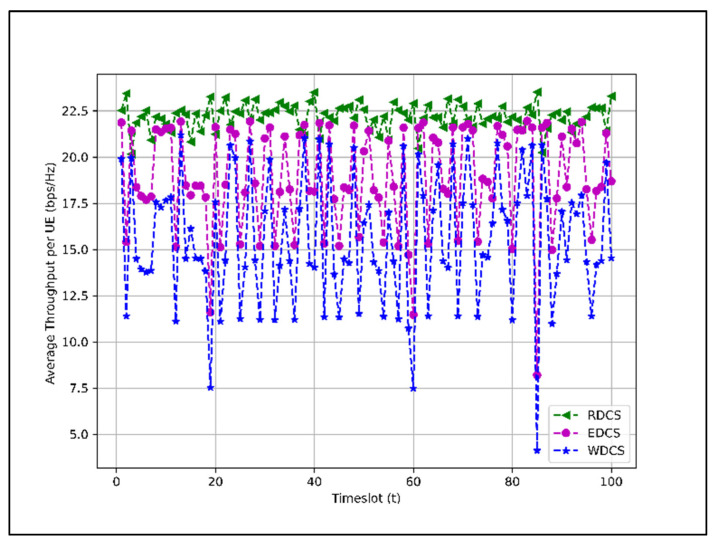
Bandwidth normalized average throughput per user (bps/Hz) for 100 timeslots.

The average throughput (bandwidth normalized) per user using schemes RDCS, EDCS, and WDCS is shown in Figure 12 for 100 timeslots.

In Figure 12, the average throughput per user for RDCS, EDCS, and WDCS varies from 20.15 (bps/Hz) to 23.52 (bps/Hz), 8.20 (bps/Hz) to 21.96 (bps/Hz), and 4.12 (bps/Hz) to 21.20 (bps/Hz) for different timeslots, respectively. The average throughput per user is higher for RDCS because it mainly selects $G_{1}$ candidates, and the UE slot is fully occupied. EDCS and WDCS, on the other hand, reserve slots for UEs from $G_{2}$ and $G_{3}$ and cause the slot to become free if there is no candidate from those groups in any timeslot.

## 5. Discussion

In this section, we discuss the results presented in Section 4 and show the limitations of this work.

Section 4.1. presents the performance of the dataset categorization scheme in terms of DLDC model test accuracy and BCE loss. The results presented in Figure 6 and Figure 7 show that the performance of the DLDC model, when trained for 10 training epochs, achieves a test accuracy of 96.7% and a BCE loss of 0.1355. The results presented in Figure 6 and Figure 7 show that the DLDC model is well trained. The trained DLDC model is used to categorize the dataset D based on $G_{i}$ in real time. After categorizing the dataset D, the proposed dataset combination procedures were applied.

Section 4.2. presents the performance of dataset combination systems such as RDCS, EDCS, and WDCS is presented based on the complexity analysis, number of users, fairness in user selection, and average user throughput. From the complexity analysis in Section 4.2.1, the complexity of RDCS, EDCS, and WDCS is the same, i.e., $O\left(TN_{a}\right)$. The user selection results shown in Figure 8, Figure 9 and Figure 10 indicate that the RDCS is likely to fully occupy the slot of UEs since there is no preference level in UE selection, while in the case of the EDCS and WDCS, the full occupancy of the available UE slot is not guaranteed. The result of fairness in UE selection in Figure 11 shows that the EDCS performs better in selecting UEs from groups $G_{2}$ and $G_{3}$ since it tries to select UEs from each group with the same proportion. Fairness in selecting UEs from $G_{1}$ is lower for the EDCS because the number of UEs in $G_{1}$ is generally higher. If the number of UEs in each group were the same, the fairness in selecting UEs from each group would be the same under EDCS. The average user throughput is shown in Figure 12. From this, it can be seen that RDCS is the best approach in terms of throughput compared to the other two schemes because it selects $G_{1}$ UEs as they are more in number and there is no preference for selection. Based on the results of fairness in selecting UEs, EDCS is the best approach, while in the case of average system throughput, RDCS is the best approach.

Although EDCS and WDCS provide fairness in selecting the UEs with medium/bad channel. Some of the limitations of the proposed method are:

- In EDCS and WDCS, UEs are selected based on $G_{i}$. Therefore, UEs selected under one group for data transmission in one time instance may belong to a different group in another time instance. However, in such a scenario, the UEs that have already had the opportunity to transmit data in the previous time instance may be preferred over the UEs of the same group that have not yet had the opportunity to transmit data.
- If there are no UEs in any of the groups during a time instance under EDCS and WDCS, there will be unused UE slots in those groups during that time instance, which may affect the overall performance of the system.

## 6. Conclusions

In this paper, a novel dataset categorization and dataset combination method is proposed to ensure a fair selection of UEs for data packet transmission. For dataset categorization, a 2D CNN-based DLDC scheme is proposed to categorize the dataset into three different groups, namely, $G_{1}$, $G_{2}$, and $G_{3}$, based on the radio channel status. The DLDC model was trained with 0.07 M 2D datasets of size 12 × 3. The simulation results show that our proposed DLDC model achieves an average test accuracy of 96.7% and BCE loss of 0.1355. Based on the results of the DLDC scheme, it is fed into the systems for combining datasets with schemes such as RDCS, EDCS, and WDCS.

The simulation results for combining datasets show that EDCS is better for selecting UEs of groups $G_{2}$ (medium channel capacity) and $G_{3}$ (poor channel capacity) because the fairness factor for $G_{2}$ and $G_{3}$ is comparatively higher in this scheme as it tries to select UEs of groups $G_{1}$, $G_{2}$, and $G_{3}$ with the same preference. RDCS, on the other hand, performs better in terms of overall system throughput as it selects most UEs from $G_{1}$ (good channel capacity) as they are larger in number. From the results, we can conclude that the dataset generated by EDCS is an ethical dataset since it tries to select UEs from $G_{1}$, $G_{2}$, and $G_{3}$ with the same preference.

## Figures and Tables

**Table 1:** Simulation parameters for generating dataset.

Simulation Parameters	Value
UEs Requesting for Connection ($N_{a}$)	12
Good Channel Condition	SIR (dB) ≥ 33
Medium Channel Condition	27 ≤ SIR (dB) < 33
Bad Channel Condition	SIR (dB) < 27
Average SIR	33 dB
Channel Variation Distribution	Rayleigh distribution
Channel Capacity	$B\times log_{2}\left(1+SIR\right)$
Total Timeslot	0.1 M

**Table 2:** Detailed structure of DLDC with four convolutional layers.

Layer Name	Input Size	Output Size	Filter Size
Conv2D_1	12 × 3, 1	12 × 3, 12	2 × 2, 12
Conv2D_2	12 × 3, 12	12 × 3, 8	2 × 2, 8
Conv2D_3	12 × 3, 8	12 × 3, 4	2 × 2, 4
Conv2D_4	12 × 3, 4	12 × 3, 1	2 × 2, 1

**Table 3:** Simulation parameters for training DLDC model.

Simulation Parameters	Values
Training Size	(70,000, 12, 3, 1)
Validation Size	(20,000, 12, 3, 1)
Testing Size	(10,000, 12, 3, 1)
Number of 2D CNN Layer	4
Number of Features	3
Number of Label	3
Batch Size	32
Learning Rate	0.0001
Epoch	1–10

**Table 4:** Complexity comparison of dataset combination schemes.

Algorithm	Complexity
RDCS	$O\left(TN_{a}\right)$
EDCS	$O\left(TN_{a}\right)$
WDCS	$O\left(TN_{a}\right)$

*T* is the total timeslot.

## Data Availability

Not applicable.
